# Liver-Directed AAV8 Booster Vaccine Expressing *Plasmodium falciparum* Antigen Following Adenovirus Vaccine Priming Elicits Sterile Protection in a Murine Model

**DOI:** 10.3389/fimmu.2021.612910

**Published:** 2021-06-23

**Authors:** Mohammad Shahnaij, Mitsuhiro Iyori, Hiroaki Mizukami, Mayu Kajino, Iroha Yamagoshi, Intan Syafira, Yenni Yusuf, Ken Fujiwara, Daisuke S. Yamamoto, Hirotomo Kato, Nobuhiko Ohno, Shigeto Yoshida

**Affiliations:** ^1^ Laboratory of Vaccinology and Applied Immunology, Kanazawa University School of Pharmacy, Kanazawa University, Kanazawa, Japan; ^2^ Division of Gene Therapy, Jichi Medical University, Shimotsuke, Japan; ^3^ Department of Parasitology, Faculty of Medicine, University of Hasanuddin, Makassar, Indonesia; ^4^ Division of Histology and Cell Biology, School of Medicine, Jichi Medical University, Shimotsuke, Japan; ^5^ Division of Medical Zoology, Department of Infection and Immunity, Jichi Medical University, Shimotsuke, Japan

**Keywords:** human adenovirus serotype 5, adeno-assoicated virus, AAV8, *Plasmodium falciparum* circumsporozoite protein, malaria, vaccine

## Abstract

Hepatocyte infection by malaria sporozoites is a bottleneck in the life-cycle of *Plasmodium* spp. including *P. falciparum*, which causes the most lethal form of malaria. Therefore, developing an effective vaccine capable of inducing the strong humoral and cellular immune responses necessary to block the pre-erythrocytic stage has potential to overcome the spatiotemporal hindrances pertaining to parasite biology and hepatic microanatomy. We recently showed that when combined with a human adenovirus type 5 (AdHu5)-priming vaccine, adeno-associated virus serotype 1 (AAV1) is a potent booster malaria vaccine vector capable of inducing strong and long-lasting protective immune responses in a rodent malaria model. Here, we evaluated the protective efficacy of a hepatotropic virus, adeno-associated virus serotype 8 (AAV8), as a booster vector because it can deliver a transgene potently and rapidly to the liver, the organ malaria sporozoites initially infect and multiply in following sporozoite injection by the bite of an infected mosquito. We first generated an AAV8-vectored vaccine expressing *P. falciparum* circumsporozoite protein (PfCSP). Intravenous (i.v.) administration of AAV8-PfCSP to mice initially primed with AdHu5-PfCSP resulted in a hepatocyte transduction rate ~2.5 times above that seen with intramuscular (i.m.) administration. This immunization regimen provided a better protection rate (100% sterile protection) than that of the i.m. AdHu5-prime/i.m. AAV8-boost regimen (60%, *p* < 0.05), i.m. AdHu5-prime/i.v. AAV1-boost (78%), or i.m. AdHu5-prime/i.m. AAV1-boost (80%) against challenge with transgenic PfCSP-expressing *P. berghei* sporozoites. Compared with the i.m. AdHu5-prime/i.v. AAV1-boost regimen, three other regimens induced higher levels of PfCSP-specific humoral immune responses. Importantly, a single i.v. dose of AAV8-PfCSP recruited CD8^+^ T cells, especially resident memory CD8^+^ T cells, in the liver. These data suggest that boost with i.v. AAV8-PfCSP can improve humoral and cellular immune responses in BALB/c mice. Therefore, this regimen holds great promise as a next-generation platform for the development of an effective malaria vaccine.

## Introduction

Malaria remains an important cause of global morbidity and mortality, predominantly in infants and young children in sub-Saharan Africa. Numerous efforts have been made to develop an effective malaria vaccine. The most clinically advanced malaria vaccine to date, RTS,S/AS01 (also known as Mosquirix™), is a protein-in-adjuvant component vaccine based on the *Plasmodium falciparum* circumsporozoite protein (PfCSP), which targets the pre-erythrocytic parasite stage and has been partially successful in human clinical trials. However, a phase III clinical trial in sub-Saharan Africa found that RTS,S/AS01 has limited efficacy (18%–26% in infants) in the first year after vaccination, and that its protection level wanes rapidly, dropping to almost zero in the fourth year after vaccination (1, 2).

The development of an effective malaria vaccine has been impeded by two major spatiotemporal factors: liver microanatomy and parasite biology. Within 30 minutes of *Plasmodium* sporozoites entering a new host after it has been bitten by a *Plasmodium*-infected female mosquito, the parasites reaching the liver invade hepatocytes. Later it undergo exoerythrocytic schizogony, and are subsequently released into the circulation as thousands of blood-stage merozoites. Therefore, to be effective, a pre-erythrocytic vaccine should induce a robust cell-mediated immune response in the liver, and this response must clear the parasites or infected hepatocytes within a narrow window of 5 to 7 days after liver infection (3). CD8^+^ T cells are necessary for protection against intrahepatic malaria parasites, and viral-vectored vaccines are better at inducing CD8^+^ T cells than protein-in-adjuvant regimens (4). Recombinant adeno-associated virus (AAV) has emerged as a promising viral vector for use in the development of effective and safe vaccines due to its broad tissue tropism, non-pathogenicity, and ability to induce efficient and long-term gene expression without causing toxicity *in vivo* (5). Moreover, the low prevalence of neutralizing antibodies against the viral capsid in human sera, rapid viral uncoating, and excellent safety profile in human clinical trials underpins the position of AAVs as viral vector-based vaccination tools (6).

We recently reported that AAV1 has the potential to induce specific antibodies targeting malaria vaccine candidate antigens (e.g., PfCSP and Pfs25). It also affords durable protection in a rodent model when administered following intramuscular (i.m.) injection of an adenovirus-vectored vaccine, but only when AAV1 is administered as the booster, not as the prime (7, 8). Differences in cell entry, tissue tropism, and/or interactions with host innate immune factors among the AAV serotypes can dictate the adaptive immune responses in the host following administration of recombinant AAV (rAAV) (9). Consequently, the administration of rAAV encoding different pathogenic antigens by various delivery methods can induce immunized animals to produce varying immune responses (10–12). The strategy behind the immunization regimen tested in the present study was to generate and maintain the level of T cell-mediated immune responses in the liver to confer adequate protection by efficiently delivering the *pfcsp* gene into hepatocytes using a liver-directed AAV serotype 8 (AAV8) vaccine construct.

Thus, we performed a comparative study in BALB/c mice to evaluate the immune responses and protective efficacy induced by immunization regimens each consisting of a prime with the i.m.-delivered human adenovirus type 5 (AdHu5) vaccine and an AAV1 or AAV8 booster vaccine delivered by i.m. or intravenous (i.v.) injection. Together with the induction of strong humoral and cellular immune responses from the AAV-based booster vaccine, the introduction of a pre-erythrocytic antigen into the liver by a hepatotropic AAV8 vaccine may result in the direct induction of liver-specific cellular immune responses capable of killing malaria parasites in the liver, the organ where they develop into their exoerythrocytic form and multiply by schizogony.

## Materials and Methods

### Ethics Statement

All animal care and handling procedures were performed under the approved guidelines of the Animal Care and Ethical Review Committee of Kanazawa University (No. 22118–1) and Jichi Medical University (No. 17086-01), Japan. All efforts were made to minimize animal suffering during the experiments.

### Parasites and Animals

Female inbred BALB/c and ddY mice were obtained from Japan SLC (Hamamatsu, Shizuoka, Japan). BALB/c mice were used to assess tissue tropism, cellular immune responses, and protection from challenge infections. A transgenic *P. berghei* parasite expressing full-length PfCSP in place of PbCSP (PfCSP-Tc/Pb) was used for the protective efficacy experiments as described previously (13–15). This transgenic parasite was maintained in the Laboratory of Vaccinology and Applied Immunology, Kanazawa University. *Anopheles stephensi* mosquitoes (SDA 500 strain) were infected with the parasites by allowing them to feed on parasitized 6-week-old ddY mice.

### Recombinant Viral-Vectored Vaccines

AAV1 and AAV8 expressing luciferase (AAV1-Luc or AAV8-Luc, respectively) were generated as described previously (16, 17). AdHu5-PfCSP-G, AAV1-PfCSP-G, and AAV8-PfCSP-G were also generated as described previously (7, 16). Briefly, the codon-optimized gene cassette encoding a GPI anchor-lacking full-length PfCSP (Leu19-Val377) gene from *P. falciparum* 3D7 strain was fused between the mouse IgGκ signal peptide and the membrane anchor sequence of the vesicular stomatitis virus (VSV)-G protein to direct localization and efficient translocation of the antigen to the cell membrane, respectively. The posttranscriptional regulatory element from woodchuck hepatitis virus (*wpre*) was then inserted into the gene cassette to enhance the expression of PfCSP protein in mammalian cells. The resulting gene construct was inserted into pAd/PL-DEST (Invitrogen, Carlsbad, CA, USA) under the control of a strong synthetic CAG promoter sequence. The adenovirus was produced (15), purified, and titrated using the Fast-Trap Adenovirus Purification and Concentration Kit (Millipore, Temecula, CA, USA) and the Adeno-X™ Rapid Titer Kit (Clontech, Palo Alto, CA, USA) according to the manufacturers’ protocols.

In the case of the AAV vectors, the gene construct was introduced into pAAV-MCS under the control of a universal cytomegalovirus (CMV) promoter sequence to construct pAAV-CMV-sPfCSP2-G(+). These plasmids were used to produce the AAV1-PfCSP-G and AAV8-PfCSP-G constructs by transfecting HEK293 cells, as described elsewhere (11, 12). AdHu5-PfCSP-G, AAV1-PfCSP-G, and AAV8-PfCSP-G were re-named AdHu5-PfCSP, AAV1-PfCSP, and AAV8-PfCSP, respectively, in the present study.

### *In Vivo* Bioluminescent Imaging

AAV1-Luc and AAV8-Luc were injected into the right tibialis anterior muscles or tail veins of the BALB/c mice (*n* = 3; 1.0 × 10^11^ plaque-forming units (pfu)/mouse) on day 0. The animals were anesthetized with a ketamine (100 mg/kg)/xylazine (10 mg/kg) mixture and, after 10 minutes, D-Luciferin (15 mg/ml; OZ Biosciences, Marseille, France) was administered intraperitoneally (i.p.) (150 µl/mouse) at the appropriate time points. On days 8 or 224, the sacrificed mice from this experiment were dissected to measure liver bioluminescence. Luciferase expression in their livers (and whole bodies) was detected using the IVIS^®^ Lumina LT *in vivo* imaging system (PerkinElmer, Waltham, MA, USA) as described previously (15, 16). The accumulated emissions were calculated, and a color heatmap was used to show the expression intensities. The visible *in vivo* luminescent images in the mice represent the total flux of photons in photons/second/cm^2^ (p/s/cm^2^) in the region of interest (ROI). The measured signal intensities are represented by radiance (p/s/cm^2^/sr), a value that refers to the number of photons per second that leave a square centimeter of tissue and radiate into a solid angle of one steradian (sr).

### Immunoblotting

HEK293T or Hepa1-6 cells (4.0 × 10^4^ cells/well) were transduced with AdHu5-PfCSP at a multiplicity of infection (MOI) of 3, with AAV1-PfCSP (MOI = 10^5^), or with AAV8-PfCSP (MOI = 10^5^) after seeding onto 48-well plates. The cell lysates collected using Laemmli buffer at 48 h post-infection were electrophoresed on 10% sodium dodecyl sulfate polyacrylamide (SDS-PAGE) gels under reducing conditions. Proteins were transferred onto Immobilon FL^®^ PVDF membranes (Merck Millipore, Tokyo, Japan). The resulting blots were blocked in 5% skim milk (Wako Chemical Inc., Tokyo, Japan) in PBS containing 0.1% Tween 20 (PBS-T). Blots were probed with the anti-PfCSP 2A10 monoclonal antibody (mAb) in 5% skim milk/PBS-T. After PBS-T washing, each membrane was probed with goat anti-mouse IgG conjugated to IRDye 800cw (Rockland Immunochemicals, Limerick, PA, USA) diluted in 5% skim milk. The membrane was visualized using an Odyssey infrared imager (LI-COR, Lincoln, NE, USA).

### Immunofluorescence Assay

HEK293T cells were transduced with AdHu5-PfCSP (MOI = 3), AAV1-PfCSP (MOI = 10^5^), or AAV8-PfCSP (MOI = 10^5^) on an 8-well chamber slide. At 48 h post-infection, the samples were treated with ice-cold 100% methanol (permeabilized) or 4% paraformaldehyde (non-permeabilized) for 15 min. The samples were then blocked with 10% normal goat serum (NGS) in PBS and incubated with 2A10 mAb in 10% NGS/PBS at room temperature. After three PBS washes, the samples were incubated with fluorescein isothiocyanate (FITC)-conjugated goat anti-mouse IgG (Invitrogen, Carlsbad, CA, USA). After PBS washing, the slide was mounted with a drop of VECTASHIELD™ containing 4′, 6-diamidino-2-phenylindole (DAPI; Vector Laboratories, Burlingame, CA, USA). All micrographs were acquired by BZ-X710 fluorescence microscopy (Keyence Corp., Tokyo, Japan).

### Immunization and Parasite Challenge

Mice were immunized with an i.m. injection of 5 × 10^7^ pfu AdHu5-PfCSP into the musculus tibialis followed by a booster with 1 × 10^11^ vector genome (vg) per mouse of either AAV1- or AAV8-PfCSP administered i.m. or i.v. into the tail vein with a 6-week interval. Control mice received 100 µl of endotoxin-free PBS. Six weeks after the last immunization, the mice were challenged with an i.v. dose of 500 PfCSP-Tc/Pb sporozoites per mouse suspended in RPMI-1640 media (Gibco, Life Technologies, Tokyo, Japan). Infections were monitored (on days 4 to 14) using the Giemsa-stained thin blood smears obtained from the tail. At least 20 fields (magnification: ×1,000) were examined before a mouse was deemed to be malaria-infection negative. Protection was defined as the complete absence of blood-stage parasitemia on day 14 after challenge.

### Enzyme-Linked Immunosorbent Assay

The PfCSP-specific antibody titers in the sera collected from the tail vein blood samples of the immunized mice, which were taken 1 day before the booster dose and sporozoite challenge, were measured by ELISA as previously described (7). Briefly, Costar^®^ EIA/RIA polystyrene plates (Corning Inc., NY, USA) pre-coated with *Escherichia coli*-produced recombinant PfCSP (400 ng/well) were blocked with 1% bovine serum albumin (BSA) in PBS and then incubated with the serially diluted serum samples or with the negative or the positive controls (2A10 mAb). An anti-mouse IgG conjugated with horseradish peroxidase (HRP) (Bio-Rad Lab Inc., Tokyo, Japan) was used as the secondary antibody. The endpoint titer was expressed as the reciprocal of the last dilution that produced an optical density (O.D.) at 414 nm of 0.15 U above the values of the negative controls (<0.1). All mice used in the experiments were seronegative before immunization.

### Liver DNA Isolation and Quantitation

Whole livers were obtained from the vaccine-protected mice in the challenge study promptly after the animals were sacrificed. Each liver was placed in a 5-ml plastic tube containing 4.0 ml of ALT buffer (Qiagen, Valencia, CA, USA), and then homogenized at 2,500 rpm for 3.5 min using a μT-12 bead crusher (Tatitec, Saitama, Japan). Total DNA was isolated from 100-µl aliquots of the resulting homogenates using a DNA isolation kit (Qiagen) in accordance with the manufacturer’s instructions. A quantitative analysis of the DNA was performed by qPCR with SYBR Green Premix Ex Taq (Takara, Tokyo, Japan). The oligonucleotide primers used for qPCR are shown in **Supplemental Table 1**. pAAV-CMV-sPfCSP2-G(+) plasmid DNA was used to generate a standard curve for the qPCR assays targeting the *pfcsp* gene sequences. A *C*_t_ cutoff was determined for each assay, whereby any well with a *C*_t_ value ≥ the mean *C*_q_ of the 10^3^ standard was omitted from the analysis because it would lie outside of the linear range of the assay. The fit-points method for absolute quantification was used for the analysis, and the noise band and threshold were set to Auto (18).

### Immunohistochemistry on the Liver Sections

Whole livers from the vaccine-protected mice in the challenge experiments were obtained after perfusion with 4% paraformaldehyde in 0.1M phosphate buffer under anesthesia. The liver tissues were subsequently immersed in the same fixatives, and 8-µm-thick cryosections were obtained following infiltration with 30% sucrose in PBS. The sections were heated at 95°C for 10 min in 0.5% Immunosaver (Nisshin EM, Tokyo, Japan), treated with 100% methanol for 10 min, and incubated overnight at 4°C in PBS containing 2.5% BSA and the 2A10 mAb. Some sections from the same mice were incubated in 2.5% BSA in PBS without 2A10 mAb for use as negative controls. After washing, all the sections were incubated in PBS containing Alexa 488-conjugated donkey anti-mouse IgG and 2.5% BSA for 3 h at room temperature. Sections were observed by light microscopy after coverslipping with VECTASHIELD™ mounting medium (Vector Laboratories, Burlingame, CA, USA) containing 4’,6-diamidino-2-phenylindole (DAPI).

### Liver-Resident Memory CD8^+^ T Cells

Mice were sacrificed 2 weeks after the i.v. administration of AAV8-PfCSP (1.0 × 10^9^, 1.0 × 10^10^, or 1.0 × 10^11^ vg) or PBS into their tail veins. After being perfused with buffer 2 (66.74 mM NaCl, 6.71 mM KCl, 6.31 mM CaCl_2_, 100 mM HEPES, 0.226 mM BSA) containing collagenase type IV (0.53 mg/ml, Sigma-Aldrich, St. Louis, UK), the livers were harvested and homogenized using frosted glass to generate single-cell suspensions. The cells were passed through a 100-µm mesh, resuspended in 35% Percoll/PBS, and centrifuged at 500 ×*g* for 20 min at room temperature. The red blood cells were subsequently lysed. Spleen cells were filtered through a 40-µm mesh and the buffy coat layer resulting from density gradient centrifugation with Histopaque^®^-1083 (Sigma-Aldrich) was collected. After the number of cells in the pellets and supernatants, excluding debris, was counted, the cells were antibody-stained in the presence of TruStain FcX™ Ab. The following antibodies and tetramer were used to stain the liver T_RM_ cells: CD45-FITC, CD8β-PerCP-Cy5.5, CD44-Brilliant Violet 510™, CD62L-APC, CD69-Brilliant Violet 421™, CXCR6 (CD186)-PE-Cy7, KLRG1 (MAFA)-APC-Cy7, and PE-conjugated PfCSP tetramer (the H-2K^d^-restricted PfCSP NYDNAGTNL epitope was provided by the National Institutes of Health Tetramer Core). The cells were washed and flow cytometrically analyzed using BD FACSVerse (the gating strategy is shown in **Figure S1**). The number of leukocytes per gram of tissue was calculated based on the percentage of CD45^+^ cells.

### Statistical Analyses

Statistical analyses were performed in Prism version 7.0a (GraphPad Software Inc., La Jolla, CA, USA) and RStudio. Depending on the data distribution, a Student’s t-test or Mann–Whitney rank test was used for comparing two groups. To assess the differences among the immunization groups, a Kruskal–Wallis test with Dunn’s correction for multiple comparisons or Tukey’s multiple comparison was used. All ELISA end-point titers were log_10_-transformed before analysis. The infection protection level was analyzed using Fisher’s exact test. A *p*-value of <0.05 was considered statistically significant.

## Results

### AAV8 Exhibits a Stronger Luciferase Expression Profile Than AAV1

**Figure 1:**
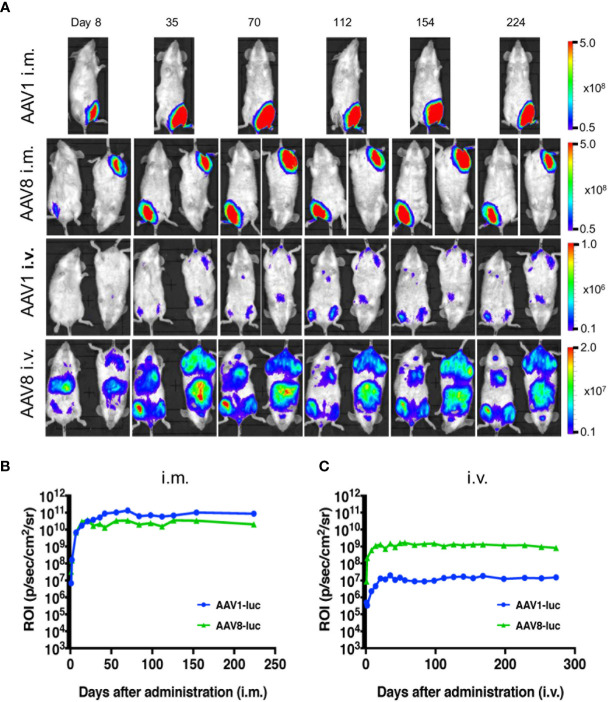
Comparison of long-term transgene expression between AAV1-Luc and AAV8-Luc. **(A)** AAV1-Luc or AAV8-Luc was injected into the right medial thigh muscles or tail veins of mice (n = 2; 1.0 × 10^11^ vg/mouse) on day 0. Luciferase expression at different time points was detected using the IVIS Lumina LT Series III *in vivo* imaging system. The heatmap images visible in the mice represent the total flux of photons (p/s/cm^2^) in the area of interest. Rainbow scale ranges are expressed in radiance (p/s/cm^2^/sr. **(B, C)** The mean total flux of photons is shown as the region of interest (ROI) from day 0 to day 224 after i.m. **(B)** or i.v. administration **(C)** AAV-Luc administration.

**Figure 2:**
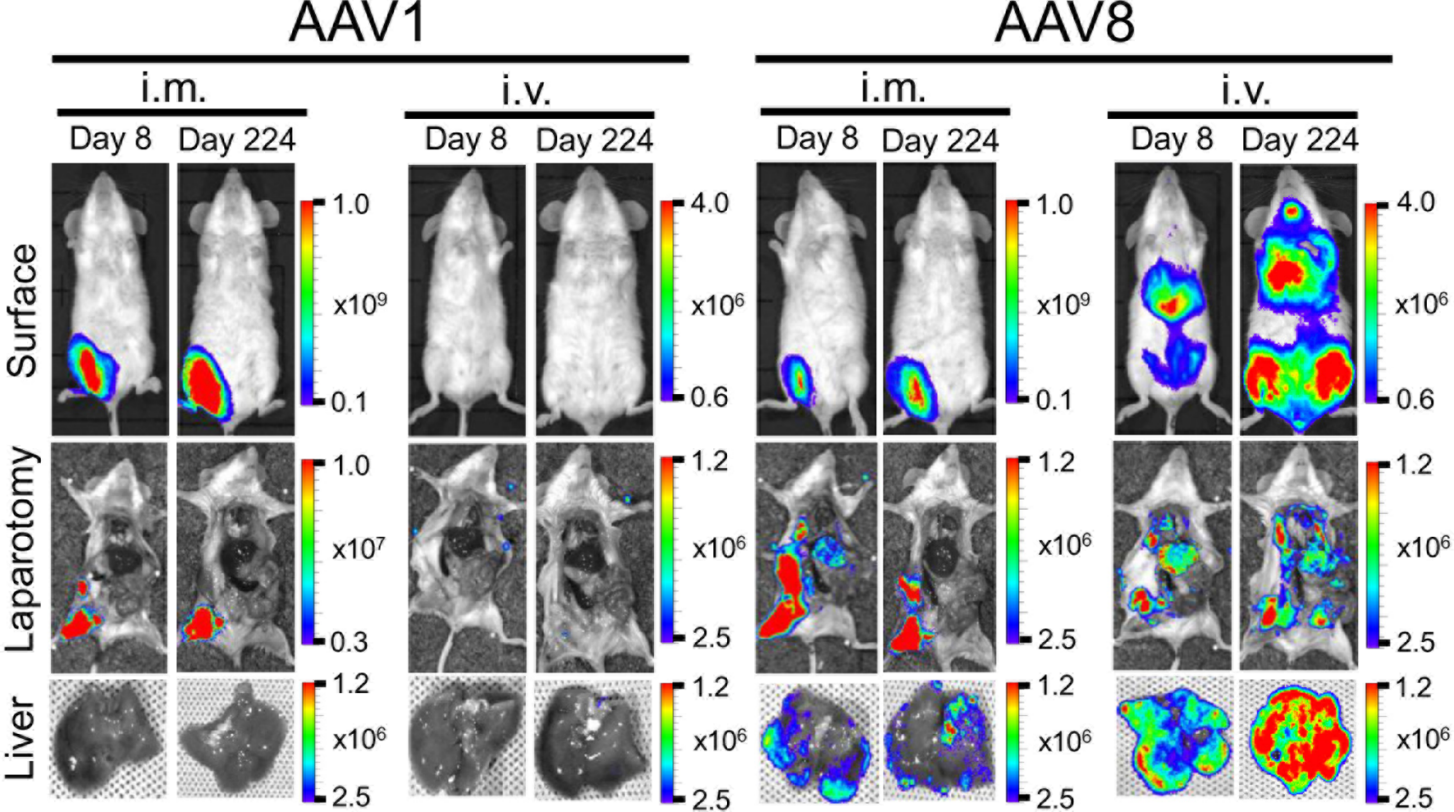
Comparison of luciferase expression in the liver between AAV1-Luc and AAV8-Luc. Luciferase expression in the liver on day 8 or day 224 was detected using the IVIS Lumina LT Series III *in vivo* imaging system. AAV1-Luc or AAV8-Luc was injected into the right medial thigh muscles or tail veins of mice (n = 2; 1.0 × 10^11^ vg/mouse) on day 0. On days 8 and 224, mice were dissected 15 min after D-luciferin was administered i.p. and luciferase expression in the liver was measured. The heatmap images in the mice represent the total flux of photons (p/s/cm^2^) in the area of interest. Rainbow scale ranges are expressed in radiance (p/s/cm^2^/sr). The mean total flux of photons is shown as the region of interest on days 8 and 224 after AAV-Luc administration with the indicated serotype and route.

Among the wide range of AAV serotypes, AAV1 and AAV8 are the two best options for producing an efficient, durable transfer and expression of the transgene in the desired tissue (19, 20). Previous *in vivo* bioluminescence imaging system (IVIS) studies have revealed that the AAV8 vector is highly hepatotropic after i.v. administration, whereas AAV1 produces an intense expression in skeletal muscle after its i.m. administration (21, 22). Therefore, we compared the expression profiles of these two promising serotypes following either i.m. or i.v. administration of a standard dose of 10^11^ vg/mouse. The resulting luminescence was robust; it gradually increased from day 0 to day 7, plateaued within 10 days, and persisted for over 224 days. The i.m. administration of AAV1-Luc or AAV8-Luc induced extensive luciferase expression (10^11^ p/s/cm^2^/sr) in the right medial thigh muscles (**Figure 1A**). When AAV1 or AAV8 were i.m. administered, the luciferase activity levels did not significantly differ between them. Conversely, following i.v. administration of these vectors, the luciferase expression level transduced by AAV8 was ~100 times higher than that transduced by AAV1 (**Figures 1B, C**). Consistent with the results from a previous study, luciferase expression (10^8^ p/s/cm^2^/sr) was also observed in the liver after i.m. administration of AAV8-Luc although the intensity was not as higher as i.v. route of delivery. Unlike in C57BL/6 mice, where it was reported that after luciferase was initially expressed in the livers of mice receiving i.m. AAV8-Luc, the luciferase levels gradually became undetectable (23), we found that luciferase expression in our BALB/c mice persisted for more than 224 days post-administration (**Figures 1**, **2**).

Moreover, the IVIS imaging results for the livers collected from mice on day 8 or day 224 post-administration of AAV-Luc showed that systemic administration of AAV8 induced ~24–43 times better liver luciferase enzyme activity when compared with i.m. administration (**Figure 2**). In the cases of i.v. or i.m. administration of AAV1, no trace of luciferase activity (detection limit ~6.0 × 10^3^ p/s/cm^2^/sr) was observed in the liver. The high levels of hepatic- and muscular-directed transgene transduction and expression we observed after a single dose of the AAV vector was administered support the superiority of AAV8 over AAV1.

### AAV Construction and *In Vitro* Transduction Efficiency in Mammalian Cell Lines

**Figure 3:**
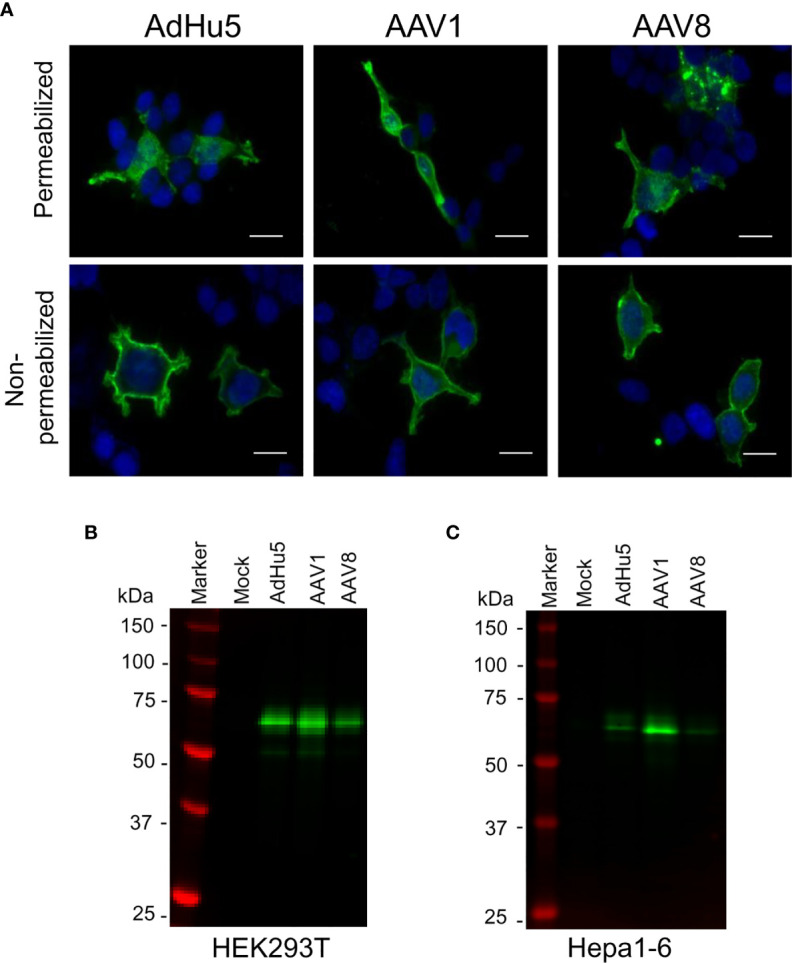
PfCSP expression in cells transduced with AAV-PfCSP. **(A)** Cellular localization of PfCSP expression in transduced cells as assessed by an immunofluorescence assay. HEK293T cells were transduced with AdHu5-PfCSP (MOI = 3), AAV1-PfCSP (MOI = 10^5^), or AAV8-PfCSP (MOI = 10^5^). After 48 h, the cells were fixed with methanol (permeabilized) or paraformaldehyde (non-permeabilized), and then treated with 2A10 mAb followed by FITC-conjugated anti-mouse secondary antibody and simultaneous staining with 4′, 6-diamidino-2-phenylindole (DAPI; blue). Original magnification, 1,000. Bars = 20 µm. **(B, C)** Detection of PfCSP in HEK293T **(B)** or Hepa1-6 **(C)** cells transduced with AdHu5-PfCSP (MOI = 3, lane 3), AAV1-PfCSP (MOI = 10^5^, lane 4), or AAV8-PfCSP (MOI = 10^5^, lane 5). Cell lysate proteins were separated by 10% SDS-PAGE and immunoblotted with 2A10 mAb.

In the liver-directed vaccine delivery experiments, we constructed an AAV8 similar to that of the AAV1-PfCSP vaccine described in our previous study (7). This construct expresses the full-length PfCSP, whereby PfCSP is anchored on the surfaces of the infected cells *via* the VSV-G protein membrane anchor, followed by the *wpre* sequence, under the control of the CMV promoter. While the CMV promoter-controlled transgene construct ensures the strong and constitutive expression of PfCSP, *wpre* enhances PfCSP gene expression. We also included VSV glycoprotein G because antigen display on the transduced cells will be more efficient in its presence. The immunofluorescence analysis of HEK293T cells transduced with AdHu5-PfCSP, AAV1-PfCSP or AAV8-PfCSP vectors revealed that PfCSP had accumulated in both the cell cytoplasmic regions and on the cell surfaces (**Figure 3A**). We next evaluated the transduction efficacy and resulting transgene expression of the virus-vectored vaccines in immortalized cell lines (HEK293T or Hepa1-6) using immunoblotting assays. Compared with AAV8-PfCSP, an identical infection dose (MOI = 10^5^) of AAV1 transduced more efficient expression in the HEK293T cell line (**Figure 3B**). This IVIS result led us to expect greater transduction and expression efficiency with AAV8-PfCSP than with the AAV1 vector in the Hepa1-6 immortalized mouse hepatic cell line. However, similar to that observed with the HEK293T cell results, a lower level of PfCSP expression was observed following AAV8-PfCSP transduction in the Hepa1-6 cell line (**Figure 3C**). This result may be explained by an improved ability of the liver tissue to take up AAV8 virus in a living organism unlike that which occurs in hepatic cell cultures.

### Administering AAV8-PfCSP by the I.V. Route After I.M. Administration of AdHu5-PfCSP Confers Complete Protection Against Transgenic *P. berghei* Expressing PfCSP

**Figure 4:**
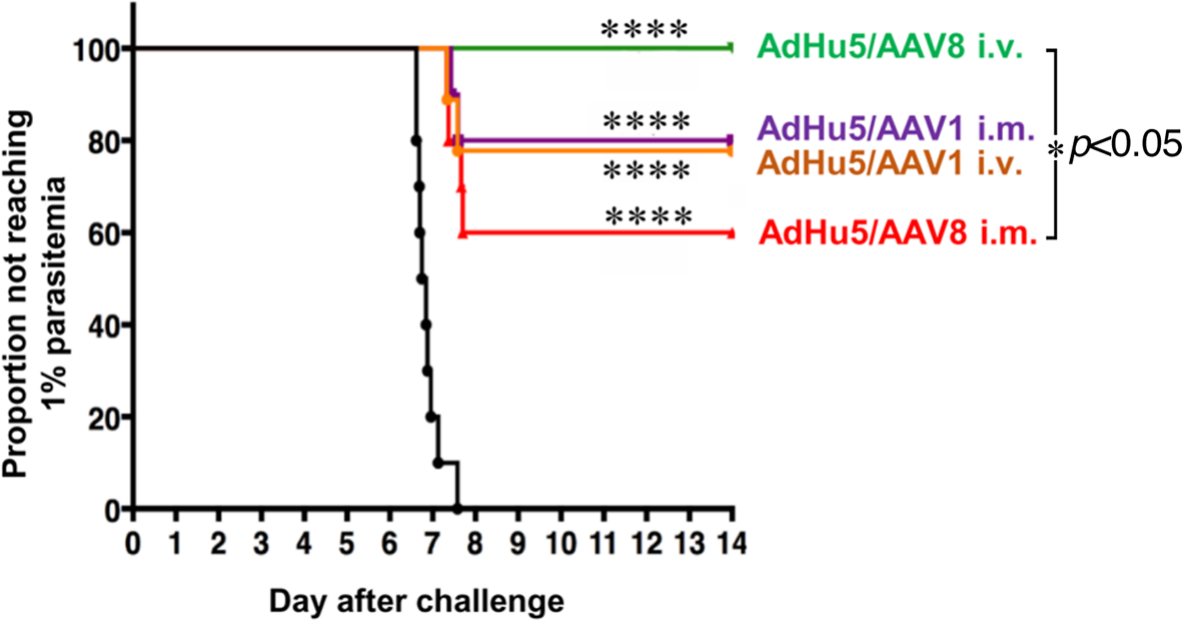
Protective efficacy of AdHu5-PfCSP prime/AAV-PfCSP boost in immunized mice. Mice (n = 9–10 per group) were immunized with an AdHu5-PfCSP prime (5 × 10^7^ pfu/mouse)/AAV-PfCSP boost (1 × 10^11^ vg/mouse) administered with a 6-week interval *via* the indicated administration routes. Six weeks after receiving booster immunizations, the mice that were challenged with 500 intravenously administered PfCSP-Tc/Pb sporozoites were checked for blood-stage infections by microscopic examination of Giemsa-stained thin smears of tail blood. Protection was defined as the complete absence of blood-stage parasitemia on day 14 post-challenge. The resulting data were statistically analyzed using the Kaplan–Meier log-rank (Mantel–Cox) test. Differences from the PBS group was assessed by a two-way ANOVA. ****p < 0.0001, and *p < 0.05.

To investigate the protective efficacy of the prime-boost regimens, mice primed by i.m. injection with the AdHu5-PfCSP vaccine followed by an i.m. or i.v. booster vaccine dose of AAV1 or AAV8 were challenged with i.v. administration of 500 transgenic PfCSP-Tc/Pb sporozoites. Consistent with our previous results (7), the i.m. AdHu5-PfCSP/i.m. AAV1-PfCSP regimen elicited an 80% protection rate. A similar level of protection (78%) was observed in mice receiving the i.m. AdHu5-PfCSP/i.v. AAV1-PfCSP regimen (**Table 1**). The immunization regimen of i.m. AdHu5-PfCSP/i.v. AAV8-PfCSP induced significantly better protection against challenge than did the immunization regimen of i.m. AdHu5-PfCSP/i.m. AAV8-PfCSP (100% vs 60%, *p* < 0.05) (**Figure 4**). The strong protective efficacy of i.m. AdHu5-PfCSP/i.v. AAV8-PfCSP against sporozoite challenge indicates that the successful elimination of sporozoites by PfCSP-specific immune responses was not only dependent on the AAV serotype but also on the administration route.

**Table 1:** Protective efficacies against sporozoite challenge by immunization with heterologous prime-boost regimens in mice^a^.

Prime (route)	Boost (route)	Protected/challenged (% protection)^b,c^
PBS	PBS	0/10 (0)
AdHu5-PfCSP (i.m.)	AAV1-PfCSP (i.m.)	7/9 (78)****
AdHu5-PfCSP (i.m.)	AAV1-PfCSP (i.v.)	8/10 (80)****
AdHu5-PfCSP (i.m.)	AAV8-PfCSP (i.m.)	6/10 (60)****
AdHu5-PfCSP (i.m.)	AAV8-PfCSP (i.v.)	10/10 (100)****

a Mice were immunized with an i.m. injection of 5 × 10^7^ pfu AdHu5-PfCSP followed by a booster with 1 × 10^11^ vg per mouse of either AAV1- or AAV8-PfCSP administered i.m. or i.v. into the tail vein at a 6-week interval. The immunized mice were challenged by i.v. administration of 500 PfCSP-Tc/Pb sporozoites. They were then screened for blood-stage infections by microscopic examination of Giemsa-stained thin smears of tail blood. Protection was defined as the complete absence of blood-stage parasitemia on day 14 post-challenge.

b Protective efficacy was calculated as described in the Materials and Methods.

c Significant difference from the control/PBS group as determined using Fisher’s exact probability test (****p < 0.0001).

### Induction of a Potent Humoral Immune Response in Mice Immunized With AdHu5-PfCSP/AAV8-PfCSP

**Figure 5:**
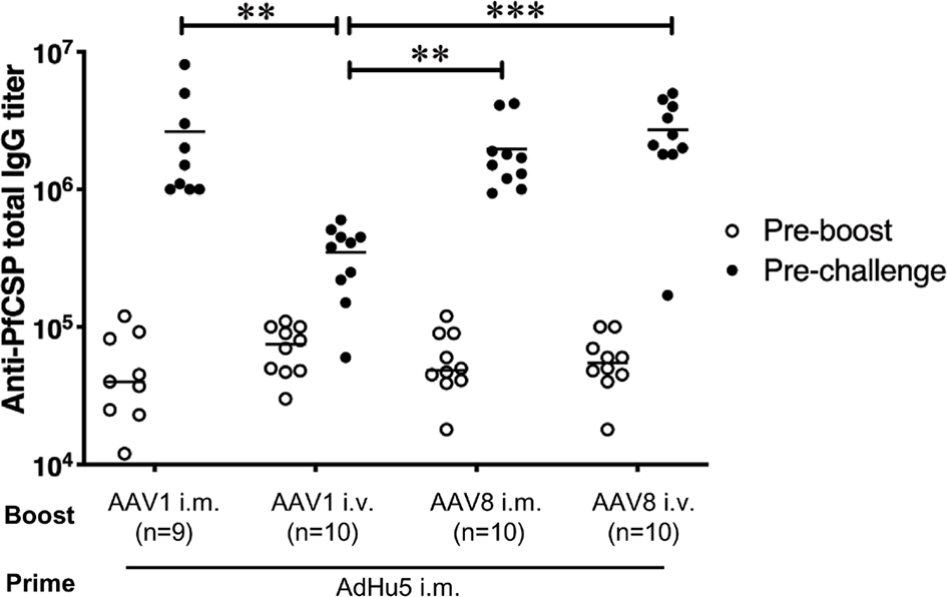
Anti-PfCSP antibody levels in mice immunized with heterologous prime-boost regimens. After immunization with i.m. AdHu5-PfCSP prime (5.0 × 10^7^ pfu/mouse)/AAV-PfCSP boost (1.0 × 10^11^ vg/mouse), mice were challenged with 500 PfCSP-Tc/Pb sporozoites. Sera were collected one day before the boost and before the sporozoite challenge from the mice used in the challenge study. Anti-PfCSP antibody titers were determined by ELISA. Bars and error bars indicate the means and SDs of the values, respectively. Between-group differences were assessed with the Kruskal–Wallis test and Dunn’s correction for multiple comparisons. ***p < 0.001 and **p < 0.01.

To compare the immunogenicity of the different vaccine regimens, we first assessed the PfCSP-specific humoral immune responses among the mouse groups receiving the various booster immunization regimens and administration routes (i.e., the PBS-treated control mice and the four groups of mice that received a booster dose with i.m. or i.v. AAV1-PfCSP or AAV8-PfCSP. All booster doses were administered 6 weeks after the mice were primed with i.m. AdHu5-PfCSP. We collected sera from tail veins 1 day before the booster injections were given and the sporozoite challenge infections commenced, and the samples were assessed by ELISA to measure the induction of PfCSP-specific humoral immune responses. All four groups of immunized mice had similar anti-PfCSP antibody levels after i.m. priming with AdHu5-PfCSP (**Figure 5**). Six weeks after the booster immunization, significant increases in the anti-PfCSP antibody titers were observed in all the test groups. Overall, the results indicate that after transduction with the booster dose, all the regimens induced high-level, PfCSP-specific humoral immune responses. A similar high-level antibody response was observed with the i.m. AAV1-PfCSP booster, a finding consistent with that reported in our previous study (7), but the immunity induced with the i.v. AAV1-PfCSP booster was significantly lower (*p*<0.01). However, the reduced humoral immune responses did not interfere with the vaccine’s protective efficacy (80% i.v. vs 78% i.m.). This finding is similar to that of the i.v. AAV8-boosted mouse group in that although a high anti-PfCSP response was observed (i.v. AAV1 vs i.v. AAV8, *p*<0.01), the AAV1 vaccines failed to elicit complete protection. Moreover, no significant difference in the total IgG level was observed between the protected and non-protected subgroups of the vaccinated mouse groups (**Figure S2**). Interestingly, i.m. AdHu5-PfCSP/i.m. AAV8-PfCSP and i.m. AdHu5-PfCSP/i.v. AAV8-PfCSP regimens produced similar total IgG levels against PfCSP with varying protective efficacies (60% i.v. vs 100% i.m., *p*<0.05).

### AAV8 Induces Superior Liver-Directed Gene Transfer Compared With AAV1

**Figure 6:**
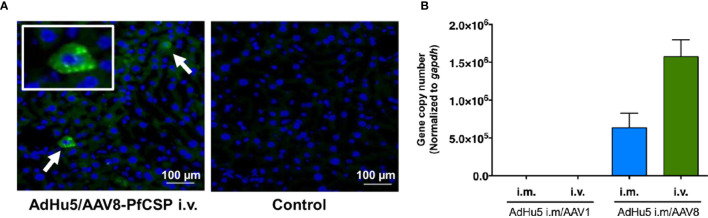
PfCSP expression profiles in the livers of the protected mice. Mice were primed with i.m. AdHu5-PfCSP (5.0 × 10^7^ pfu/mouse) and boosted with AAV-PfCSP (1.0 × 10^11^ vg/mouse) followed by a challenge with 500 PfCSP-Tc/Pb sporozoites. Whole livers were obtained from the protected mice in the challenge study. **(A)** Immunohistochemistry was performed on the liver microsections with 2A10 mAb followed by Alexa 488-conjugated anti-mouse secondary antibody (green). (**A**, left panel) Arrows show PfCSP-expressing hepatocytes. (**A**, right panel) As a negative control, a liver section from the same mouse was incubated with the solution without the mAb. Nuclei were simultaneously stained with DAPI (blue). **(B)** Quantitation of the *pfcsp* gene in the livers collected from the protected mice in the challenge experiments. The pAAV-CMV-sPfCSP2-(G+) plasmid was used to generate a standard curve with which to determine the *pfcsp* copy number in the liver. The mouse *gapdh* gene was used for normalization. ‘i.m.’ and ‘i.v.’ indicate the route of booster immunization with AAV1 or AAV8 following AdHu5 i.m. prime.

The ability of i.m. AdHu5-PfCSP/i.v. AAV8-PfCSP immunization to transduce *pfcsp* gene expression in the liver was confirmed by immunohistochemistry (**Figure 6A**). Therefore, we performed qPCR to quantify the DNA transduction levels in the livers dissected from the mice that were protected against sporozoite challenge (**Figure 6B**). The qPCR results indicated that the i.m. AdHu5-PfCSP/i.v. AAV8-PfCSP regimen was ~2.5 times better at transducing hepatocytes in the liver compared with the i.m. AdHu5-PfCSP/i.m. AAV8-PfCSP regimen. No traceable signal was detected in the liver DNA from the AAV1-immunized mouse groups.

### AAV8-PfCSP I.V. Administration Induces Liver-Resident Memory CD8^+^ T Cells

**Figure 7:**
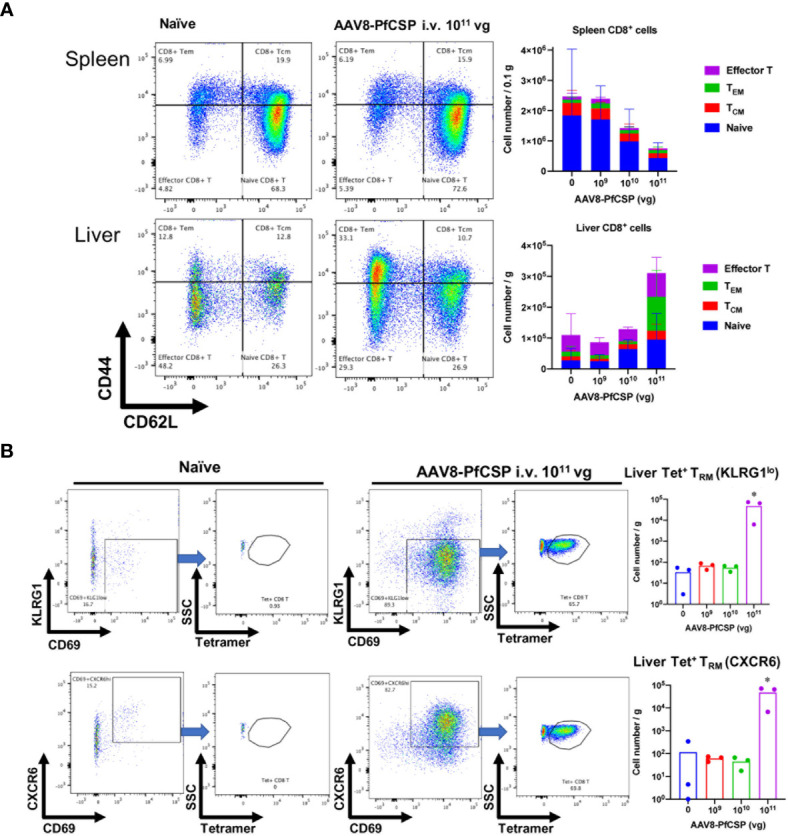
T-cell responses in the liver and spleen after i.v. immunization with AAV8. Mice were immunized with AAV8-PfCSP (n = 3 per group; 1.0 × 10^9^, 1.0 × 10^10^, or 1.0 × 10^11^ vg) or PBS *via* i.v. administration into their tail veins. After 2 weeks, the CD8^+^ T cells collected from the livers and spleens were stained as described in the Materials and Methods. Gated CD45^+^CD8^+^T-cells in livers (**A**, lower panels) or spleens (**A**, upper panels) were analyzed for CD62L and CD44 expression. The mean absolute numbers of the spleen or liver CD8^+^T cells are shown in the right-hand graphs. **(B)** Flow cytometric measurement of CD69 ^+^ and KLRG1^lo^ (**B**, upper panels) or CXCR6^+^ (**B**, lower panels) tetramer-stained, CSP-specific liver T_RM_ cells in the CD45^+^CD8^+^CD62L^lo^CD44^hi^ T_EM_ population. Mean absolute numbers of the spleen or liver PfCSP-specific CD8^+^T_RM_ cells are shown in the right-hand graphs. *p < 0.05 by one-way nonparametric ANOVA (Kruskal–Wallis) followed by the Dunn multiple comparisons test.

Antigen-expressing hepatocytes can promote the development of cytotoxic T cells in the liver (24). CD8^+^ T cell populations and their subsets were quantified to examine an immunological indicator of protection after i.v. administration of AAV8-PfCSP. Liver-resident memory T (T_RM_) cells were extensively investigated because these cells may act as sentinels against invading pathogens (25, 26). T_RM_ cells are defined as CD69^+^/KLRG1^lo^ or CD69^+^/CXCR6^+^ effector memory T (T_EM_) cells (CD8^+^CD62L^lo^CD44^hi^ cells). Two weeks after administering i.v. AAV8 to each mouse in a dose-dependent manner, the number of liver CD8^+^ T cells, especially those belonging to the T_EM_ cell population, was increased at a dose of 10^11^ vg per mouse, whereas these cells were gradually decreased in the spleen (**Figure 7A**). PfCSP-specific CD8^+^ liver T_RM_ cells (both KLRG1^lo^ and highly efficient CXCR6^+^ T_RM_ cells) were significantly induced by immunization with the standard dose of 10^11^ vg compared with the non-immunized group (**Figure 7B**). These results suggest that vaccine doses below 1.0 × 10^11^ vg/mouse are unable to efficiently recruit T cells to the liver, particularly antigen-specific CD8^+^ T_RM_ cells, 2 weeks after immunization with AAV8. This result is noteworthy because such cells may play an important role in eliminating sporozoite and/or infected hepatic cells upon parasite challenge.

## Discussion

Our recent study showed that a heterologous AdHu5-PfCSP-prime and AAV1-PfCSP-boost immunization regimen administered by the i.m. route in a murine model can elicit 80% protection against sporozoite challenge with a strong and durable PfCSP-specific humoral and cellular immune response (7). We hypothesized that the induction of local immunity in the liver could be important for preventing the exoerythrocytic stages of malaria and subsequent blood infection. Therefore, we investigated whether hepatotropic AAV8 was capable of stimulating intense transgene expression and effective T cell recruitment in the liver. We also compared the protective efficacies of i.m. or i.v. AAV1-PfCSP and AAV8-PfCSP boost regimens administered 6 weeks after i.m. AdHu5-PfCSP priming. Notably, immunization with the i.m. AdHu5-PfCSP-prime/i.v. AAV8-PfCSP-boost regimen elicited 100% protection against sporozoite challenge.

Vaccines aimed at inducing humoral and cell-mediated immunity against malaria parasites have been hindered by parasite biology and liver microanatomy (3). Successful parasite clearance in the short window of time (5 to 7 days) during the asymptomatic pre-erythrocytic malarial infection stage requires the generation and maintenance of an adequate amount of *Plasmodium*-specific humoral and cellular immune responses to confer protection (24, 27, 28). Our heterologous prime/boost immunization regimens generated a high level of PfCSP-specific antibodies in our mouse model (**Figure 5**), although the anti-PfCSP antibody titers were not correlated with protective efficacy against sporozoites (**Figure S2**). However, it has been suggested that anti-CSP antibody titers can be used as surrogate markers of protection. Therefore, such titers were previously employed to study the magnitude and duration of RTS,S/AS01 efficacy (29). Thus, the strong PfCSP-specific antibody titer induced by our two-dose AdHu5/AAV immunization strategy, especially by the i.m. AdHu5-PfCSP-prime/i.v. AAV8-PfCSP-boost regimen, is a crucial feature of translatable vaccines. Similarly, we investigated the recruitment and proliferation of CD8^+^ T cells in the liver following i.v. AAV8 administration because induced CD8^+^ T cells are reportedly required for sterile protection and are correlated with efficacy against challenge with malaria parasites (3, 30, 31). However, our current evaluation system did not address whether the protection rate was correlated with the anti-PfCSP IgG level and/or with liver-resident T cells. To elucidate the mechanism(s) underlying the protective efficacy of the i.m. AdHu5-PfCSP-prime/i.v. AAV8-PfCSP-boost regimen will require further experiments to clarify how immune responses operate in the protected and non-protected mice.

Because of the high abundance of a laminin co-receptor and a possible unknown receptor in the liver, AAV8-based vectors are very efficiently taken up by the parenchymal cells in the liver (32). Consistent with other reports, our results confirm the rapid and efficient *in vivo* transgene expression transduced by AAV8; thus, an AAV8-based vector is an attractive vector to choose for transgene delivery to the liver (3, 6, 22, 33). We found that, depending on the dose used, AAV8 could transduce as much as 90%–95% of hepatocytes in the mouse liver following its i.v. administration (34), which delivered ~2.5 times more DNA into the liver compared with i.m. administration (**Figure 6B**). Antigen expression by hepatocytes may modulate the T-cell immune response, thereby improving the clinical efficacy of rAAV (3). Our flow cytometric experiment revealed that, in a dose-dependent manner, CD8^+^ T cells, particularly T_EM_ cells and T_RM_ cells, were generated and recruited to the liver following a single dose of i.v. AAV8 (**Figure 7**). Higher efficacy against *P. falciparum* malaria is correlated with higher numbers of CXC6 T_RM_ cells in an animal model and circulating IFN-γ secreting T_EM_ cells in human recipients after immunization with attenuated *P. falciparum* sporozoites (35, 36).

We investigated the capability of i.v. AAV8-PfCSP to recruit and induce PfCSP-specific T_RM_ cells in the liver. Because liver-resident T_RM_ cells are capable of providing protection against a malaria sporozoite challenge (3, 33, 37), the observed significant increase in the number of T_RM_ cells may act to improve the protection rate. T_RM_ cells migrate to and patrol the liver sinusoids using a crawling motion and by scanning for the cognate antigen on the cell surfaces of the hepatocytes and/or the invading malaria sporozoites (37). Cytokine production (e.g., interferon-γ, IFN-γ; tumor necrosis factor α, TNF-α) and cytotoxicity marker production [e.g., granzyme B and CD107a (33)], are associated with T_RM_ cell-mediated protection against malaria, indicating that these cells may be poised to respond to immediate threats (37).

The use of both adenovirus and AAV as vaccine vehicles has been shown to be safe in human trials (38, 39). However, capsid-specific T-cell responses and pre-existing natural antibodies against these vectors from previous exposure to natural infections with them, particularly adenovirus transduction, are important considerations for their clinical application. AAV8, a relatively new isolate, has been cloned from nonhuman primate tissues, and experience with vectors based on this serotype is limited (40). However, the seroprevalence of anti-AAV8 capsid protein antibodies and cross-reactions with natural antibodies against other AAV serotypes were found to be low in human sera and, when present, they were found to have low activities (41, 42). Because a high prevalence of AdHu5 viral capsid-specific antibody was detected in African countries where malaria prevalence is high, administrating an AAV8 booster may help with maintaining the magnitude of the humoral immune response against the encoded antigen. A better priming vaccine might be achieved by the application of a different clinically tested non-immunogenic human or chimpanzee adenoviral vector [i.e., serotype 26 or serotype 63 (16)].

In summary, we exploited hepatotropic AAV8 as the booster component in a promising immunization regimen beginning with an AdHu5 priming vaccine targeting the PfCSP antigen against the malaria parasite’s liver stage in preclinical settings. AAV8 is intensely hepatotropic, and its i.v. administration induces robust expression of the *pfcsp* gene after its liver-directed delivery. Consequently, strong PfCSP-specific humoral immune responses along with high levels of CD8^+^ T cells, particularly T_RM_ cells, were generated and recruited in adequate amounts to the liver through its use. Although further studies are required to assess the involvement and durability of the T cells conferring the type of sterile protection we observed, the i.m. AdHu5-PfCSP/i.v. AAV8-PfCSP immunization regime successfully eliminated transgenic *P. berghei* parasites in sporozoite challenge infections and achieved 100% sterile protection in our BALB/c mouse model. Therefore, we propose that the i.m. AdHu5-PfCSP/i.v. AAV8-PfCSP immunization regimen has great potential for use in the development of an effective malaria vaccine.

## Data Availability Statement

The original contributions presented in the study are included in the article/**Supplementary Material**. Further inquiries can be directed to the corresponding author.

## Ethics Statement

The animal study was reviewed and approved by Animal Care and Ethical Review Committee of Kanazawa University (No. 22118–1) and Jichi Medical University (No. 17086-01).

## Author Contributions

Study concept and design: MS, MI, and SY. Acquisition of data: MS, MI, HM, MK, IY, IS, YY, KF, DY, HK, NO, and SY. Analyses and interpretation of data: MS, MI, NO, and SY. Drafting the manuscript: MS, MI, and SY. Critical revision of the manuscript for important intellectual content: MS, MI, YY, and SY. Statistical analyses: MS, and MI. Technical or material support: MS, MI, HM, DY, HK, NO, and SY. Study supervision: SY. All authors contributed to the article and approved the submitted version.

## Funding

This work was supported by a Grant-in-Aid for Scientific Research (B) (JSPS KAKENHI grant number 19H03458) and Grant-in-Aid for Scientific Research (JSPS KAKENHI grant number 18K19394) to SY, by a Grant-in-Aid for Scientific Research (C) (JSPS KAKENHI grant number 18K06655) to MI, and by a MEXT fellowship (183193) to MS.

## Conflict of Interest

SY, MI, and HM are named inventors on filed patents related to immunization with the AAV anti-malarial vaccines. These products have not been commercialized.

The remaining authors declare that the research was conducted in the absence of any commercial or financial relationships that could be construed as a potential conflict of interest.
