# Supplementary material for: Liver-Directed AAV8 Booster Vaccine Expressing Plasmodium falciparum Antigen Following Adenovirus Vaccine Priming Elicits Sterile Protection in a Murine Model
Source: Front Immunol. 2021 Jun 23;12:612910. doi: 10.3389/fimmu.2021.612910 (PMC8261234; doi:10.3389/fimmu.2021.612910)

Supplementary figure 1

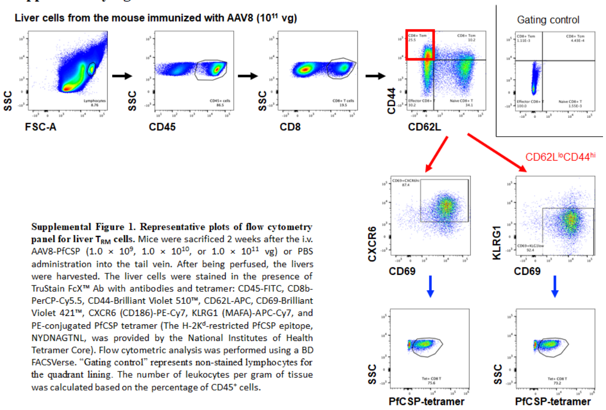

Supplemental Figure 1. Representative plots of flow cytometry panel for liver T_{RM} cells. Mice were sacrificed 2 weeks after the i.v. AAV8-PfCSP (1.0×10^9, 1.0×10^{10}, or 1.0×10^{11} vg) or PBS administration into the tail vein. After being perfused, the livers were harvested. The liver cells were stained in the presence of TruStain FcX™ Ab with antibodies and tetramer: CD45-FITC, CD8b-PerCP-Cy5.5, CD44-Brilliant Violet 510™, CD62L-APC, CD69-Brilliant Violet 421™, CXCR6 (CD186)-PE-Cy7, KLRG1 (MAFA)-APC-Cy7, and PE-conjugated PfCSP tetramer (The H-$2K^d$-restricted PfCSP epitope, NYDNAGTNL, was provided by the National Institutes of Health Tetramer Core). Flow cytometric analysis was performed using a BD FACSVerse. "Gating control" represents non-stained lymphocytes for the quadrant lining. The number of leukocytes per gram of tissue was calculated based on the percentage of $CD45^+$ cells.

Supplementary figure 1, continued

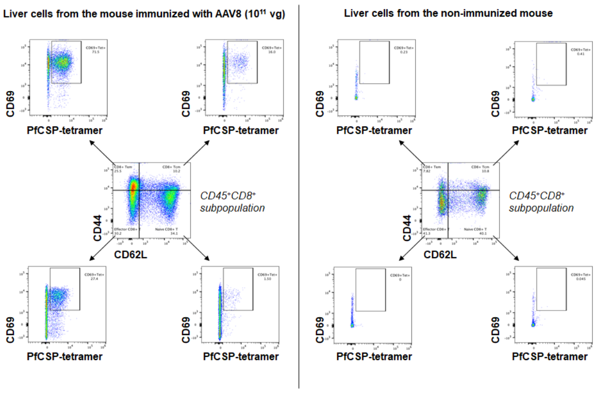

Supplement: Supplementary file 1 [file DataSheet_1.pdf]
