# Supplementary figures and images for: Liver-Directed AAV8 Booster Vaccine Expressing Plasmodium falciparum Antigen Following Adenovirus Vaccine Priming Elicits Sterile Protection in a Murine Model
Source: Front Immunol. 2021 Jun 23;12:612910. doi: 10.3389/fimmu.2021.612910 (PMC8261234; doi:10.3389/fimmu.2021.612910)

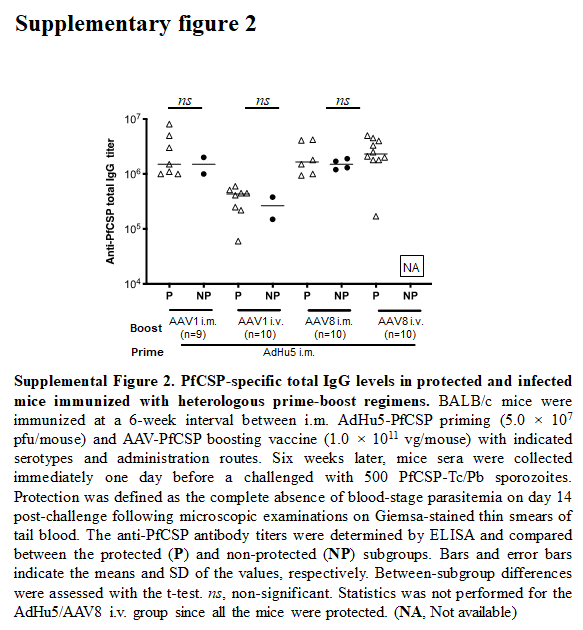

Supplement: Supplementary file 2 [file Image_1.tif]
