# Supplementary material for: Liver-Directed AAV8 Booster Vaccine Expressing Plasmodium falciparum Antigen Following Adenovirus Vaccine Priming Elicits Sterile Protection in a Murine Model
Source: Front Immunol. 2021 Jun 23;12:612910. doi: 10.3389/fimmu.2021.612910 (PMC8261234; doi:10.3389/fimmu.2021.612910)
Supplement: Supplementary file 3 [file Table_1.pdf]

Supplementary Table 1.

Oligonucleotide primer sets used to quantify the *pfcsp* gene levels in the liver. The mouse *gapdh* gene was used for normalization.

Primer Name	Primer Sequence
pGAPDH-F	5’-TGCCCCCATGTTTGTGATG-3’
pGAPDH-F	5’-TGTGGTCATGAGCCCTTCC-3’
pPfCSP-F	5’-AACAATGAAGAGCCCTCCGA-3’
pPfCSP-R	5’-ACTGCCGGGTTTGATCCTTA-3’
